# Dual-cavity feedback assisted DFB narrow linewidth laser

**DOI:** 10.1038/s41598-017-01351-w

**Published:** 2017-04-26

**Authors:** Shihong Huang, Tao Zhu, Guolu Yin, Tianyi Lan, Fuhui Li, Ligang Huang, Min Liu

**Affiliations:** 1 Key Laboratory of Optoelectronic Technology & Systems (Ministry of Education), Chongqing University, Chongqing, 400044 China; 2 State Key Laboratory on Integrated Optoelectronics, Institute of semiconductors, Chinese Academy of sciences, Beijing, China

## Abstract

Single longitudinal mode (SLM) distributed feedback (DFB) lasers with a linewidth lower than a few kHz find applications in many coherent detection systems. In this paper, we proposed and experimentally demonstrated a novel method to compress the linewidth of a SLM DFB laser by utilizing a dual-cavity feedback structure (DCFS). The DCFS first provides optical self-injection feedback to compress the laser linewidth, and then the two feedback lengths are carefully optimized to achieve SLM output via the Vernier principle and the suppression of modes overlapping between two cavities. The linewidthes of 1 MHz and 200 kHz were successfully compressed to ~2.7 and 1.5 kHz with a side mode suppression ratio of 38 and 45 dB, respectively. The stability of the DCFS output power can be controlled within ~0.21%. Our method provides a simple, effective, low cost way to achieve DFB linewidth compression, which will greatly improve the performance of coherent detection systems using DFB laser as sources.

## Introduction

It is generally known that single longitudinal mode (SLM) distributed feedback (DFB) lasers have become widely used devices due to their mature production process, small volume and low cost^1^. However, the MHz or several hundreds of kHz linewidths of most SLM DFB lasers limit their application in coherent optical systems such as optical fiber sensing; optical fiber communication; laser radar and distributed oil pipeline monitors, because the performance parameters of range; sensitivity; precision and noise, of these systems strongly depends on the linewidth span of the laser source employed^1–7^. Therefore, how to suppress the SLM DFB laser linewidth from MHz to several kHz has become a matter of great interest.

Up to now, many techniques have been used to obtain narrow SLM linewidth lasers including; short cavity distributed Bragg reflectors^8^; microfibers^9^; Rayleigh backscattering^10–12^; whispering gallery mode (WGM) resonators^13^; electrical feedback control methods^14, 15^ and optical self-injection feedback^16–21^. The electrical feedback control method can compress the laser linewidth from MHz or hundreds of kHz to a few kHz, however it usually suffers from being a complex electrical system. The frequency fluctuations of the laser have to be converted to intensity variations by a frequency discriminator such as a high finesse Fabry-Perot (FP) resonator or π-phase shifted fiber Bragg grating (FBG), and then the DFB linewidth is finally suppressed by a servo feedback control system proportional to the error signal induced by the intensity variations. Resonant optical feedback is a well-known technique to reduce laser linewidth. This technique uses a high finesse resonant cavity as a filter, effectively creating a frequency compressed light signal which is fed back into the laser itself, to suppress linewidth. Optical direct self-injection feedback is another interesting technique to compress laser linewidth by a simple external cavity structure. However, reducing the laser linewidth to 20 kHz or so from MHz using such external cavity reflection is difficult to achieve, mainly limited by spectral mode hops induced by the long external cavity.

In this paper, we constructed a simple dual-cavity feedback structure (DCFS) for the first time to compress DFB laser linewidth. For comparison, both single and dual-cavity feedback were analyzed to investigate the effects of external cavity length and external feedback rate on DFB laser linewidth compression and their longitudinal mode characteristics. Compared with the previous single-cavity feedback structure, DCFS can not only largely broaden the free-spectral-range (FSR) to achieve SLM laser oscillation easily, but also the approach reduces the linewidth from ~MHz level to several kHz with suitable feedback lengths. Self-coherent envelope linewidth detection (SCELD) is used to ensure the accuracy of the detected laser linewidth^22, 23^.

## Principle analysis

Numerous efforts have been devoted to set up the module of external self-injection feedback, which can be calculated as follow^18, 19, 24, 25^,

1 $${\rm{\Delta }}f=\frac{{\rm{\Delta }}{f}_{0}}{{[1+(1-{R}_{in})\frac{{\tau }_{e}}{{\tau }_{in}}\frac{\sqrt{\varepsilon \gamma {R}_{e}}}{\sqrt{{R}_{in}}}\sqrt{1+{\alpha }^{2}}\cos (w{\tau }_{e}+{\tan }^{-1}\alpha )]}^{2}}$$

where Δf_0_ is the linewidth of the DFB laser without feedback, τ_e_ = 2nL/c is the external round trip delaying time, c is the speed of light, n is the refractive index of the fiber core, L is the feedback length of a single-pass cavity, τ_in_ is the round trip time in the semiconductor chip, R_in_ is the facet power reflectivity of the DFB output port, Re is the external power reflectivity, which is defined as the feedback rate, α is the linewidth enhancement factor, ε is a correction factor which represents the linewidth perturbation term embedded in the feedback light induced by the external noise. In this equation, we added a parameter γ to represent the output isolation of DFB laser itself. When γ is small enough, e.g. γ = 0.0008 for 31 dB isolation in our DFB laser, a weak feedback is produced to inject back into the DFB system, and the laser linewidth is gradually compressed after multiple weak feedback circulation. This linewidth compressing phenomenon is similar to the linewidth compression by using weak Rayleigh backscattering^10–13^.

From equation (1), longer feedback length favors a narrower laser linewidth. However, a long feedback length would produce dense modes with a small FSR, due to the 1/(nL) dependence, leading to mode-hopping and obstruction of SLM operation. To solve this problem, a DCFS was introduced to broaden the FSR and suppress mode-hopping. The mode spacing in a DCFS arises from the two feedback lengths of L_1_ and L_2_ so that the FSR is given by; FSR = c/n/(L_1_ − L_2_). The FSR can be broadened to achieve SLM laser output as long as the value of ΔL = L_1_ − L_2_ is small enough. However, for certain bandwidths, the modes in two resonator cavities may overlap if ΔL = L_1_ − L_2_ is too small, and the overlapped modes will become the new side modes, thus preventing any improvement in the side-mode suppression ratio (SMSR) of the laser. Therefore, besides the broadening of FSR, the ΔL has to be carefully selected to suppress the mode overlap to achieve operation as a SLM DFB laser with high SMSR.

## Experiment and discussion of results

**Figure 1:**
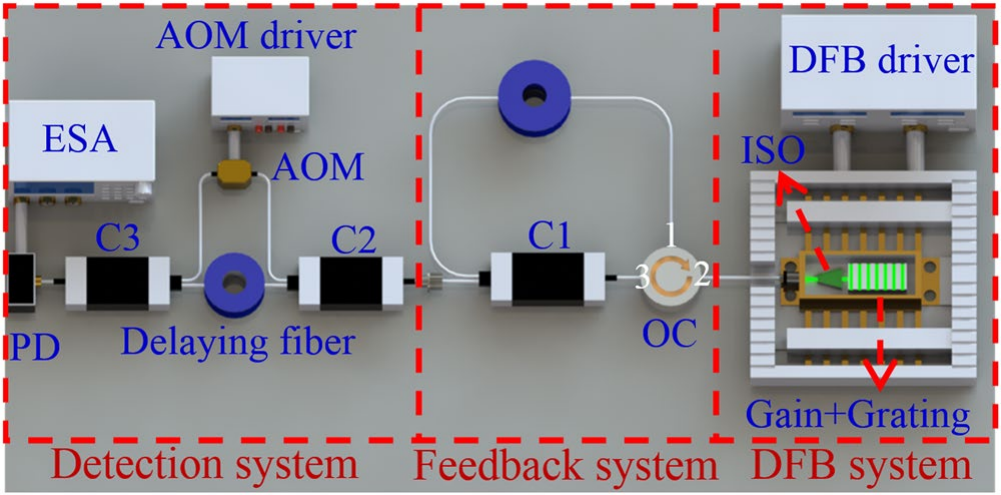
Schematic of the experimental setup. ISO: optical isolator, OC: optical circulator, C1, C2, C3: fiber optic couplers, AOM: acoustic optical modulator, PD: photodetector, ESA: electric spectrum analyzer.

Figure 1 illustrates the experimental setup with single-cavity self-injection feedback to study the relationship between the compression linewidth and the feedback rate (Re) with constant feedback length. The DFB laser works at the wavelength of 1531.13 nm with ~1 MHz linewidth, which is driven by a Model 6100 laser diode current and temperature controller (Newport Corp.), and the feedback system consists of an optical circulator (OC), a 1 × 2 fiber optic coupler (C1) and a feedback fiber. The laser linewidth is measured by our previously proposed SCELD method with 200 m delaying fiber^22, 23^.

**Figure 2:**
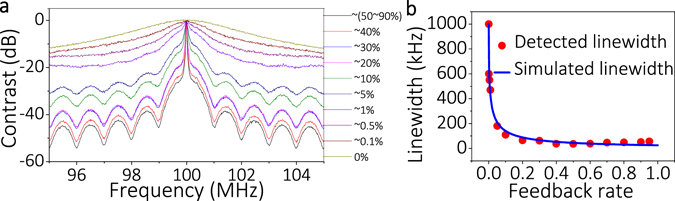
(**a**) Normalized power spectrum for different feedback rate (Re). (**b**) Corresponding fitting linewidth (red point) from (**a**) and the simulated linewidth for different feedback rate (Re) (blue curve) with Δ*f*_0_ = 1 MHz, τ_in_ = 0.0534 ns, α = 5, γ = 0.0008 (~31 dB isolation of DFB isolator), R_in_ = 0.9, τ_e_ = 2nL/c = 104.8 ns with L = 10.7 m, ε = 1.

Figure 2(a) shows the normalized power spectrum for different feedback rate with constant feedback length ~10.7 m. Red points in Fig. 2(b) show the fitting linewidths according to the power spectrum for different feedback rate in Fig. 2(a) and the blue curve in Fig. 2(b) shows the simulated linewidths from equation (1). The 5~95% feedback rate was obtained by changing the split ratio of C1 and the 0~5% feedback rate was realized by adjusting the variable optical attenuator added in the feedback cavity. In the range of 0~50% feedback rate, the higher the feedback rate is, the narrower the laser linewidth is. The experimental fitting results are consistent with the simulated results (see Fig. 2(b)). In the range of 50~95% feedback rate, since the detected power spectra change little and their linewidths almost remain the same, we only use the power spectrum with 50% feedback rate as representative data in Fig. 2(a). The linewidth should reduce as the feedback rate increases from 50 to 90% according to the simulated results, but in the experimental results it changes little or even broadens (see Fig. 2(b)) because more external noise (ε ≠ 1) would also be injected into the DFB laser, preventing laser linewidth compression with higher feedback rates. Therefore, considering the laser output power and linewidth compression, the feedback rate can be set in the range of 10~60%.

**Figure 3:**
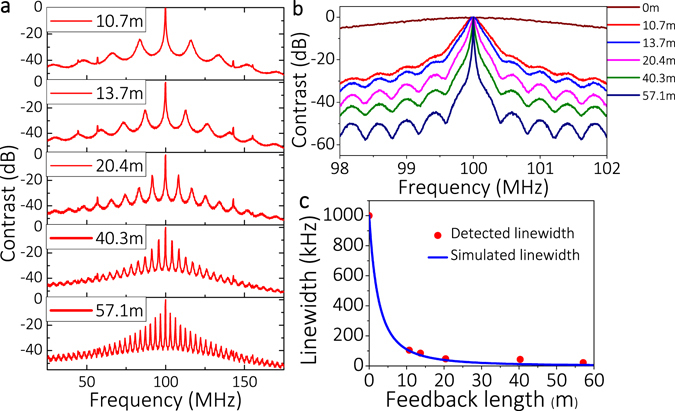
(**a**,**b**) Normalized power spectra for different feedback lengths with single-cavity feedback. (**c**) Corresponding fitting linewidth from (**b**) (red points) and the simulated linewidth for different feedback length (blue curve) with Δ*f*_0_ = 1 MHz, τ_in_ = 0.0534 ns, α = 5, γ = 0.0008 (~31 dB isolation of DFB isolator), R_in_ = 0.9, Re = 0.1, ε = 1.

To study the characteristics of output laser with different feedback length, we chose the L to be 10.7, 13.7, 20.4, 40.3, and 57.1 m in succession under 10% feedback rate (see Fig. 3(a)). It is found that the FSR decreased from 16.25 to 3 MHz, and the side mode suppression ratio (SMSR) decreased from 23 to 11 dB, which means it is easy to produce mode-hopping and more difficult to achieve stable SLM output when we increased the feedback length. However, the longer feedback length favors the linewidth compression. It is found that the linewidth is compressed from ~1 MHz to 22 kHz when L is increased from 0 m to 57.1 m (see Fig. 3(b) and (c)). It seems that laser linewidth tends to continue compressing when L is further increased to 57.1 m. However, the long external feedback length will introduce more external noise feedback at the same time. Hence, the experimental results show that the laser linewidth changes little when L varies from 20.4 to 57.1 m (see Fig. 3(c)). Therefore, the feedback length has to be chosen on the basis of linewidth compression. From the above experiments, it can be seen that single-cavity feedback structure brings the linewidth from ~1 MHz down to ~22 kHz but it is easy to produce mode hopping. Therefore, a DCFS was proposed to suppress the side mode and achieve SLM operation of the laser.

**Figure 4:**
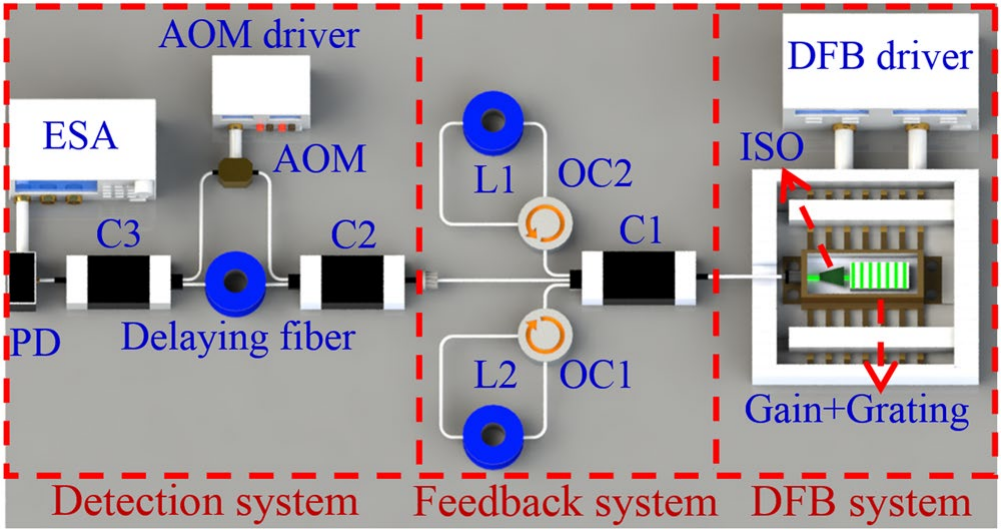
Schematic of DFB dual-cavity self-feedback structure.

Figure 4 illustrates the schematic of the DCFS. The output of the DFB laser is injected into the input pigtail of a 1 × 3 coupler. Two of three output pigtails of the 1 × 3 coupler are connected to two feedback cavities through two optical circulators. The third pigtail was connected to the SCELD-based detected system with 500 m delaying fiber.

**Figure 5:**
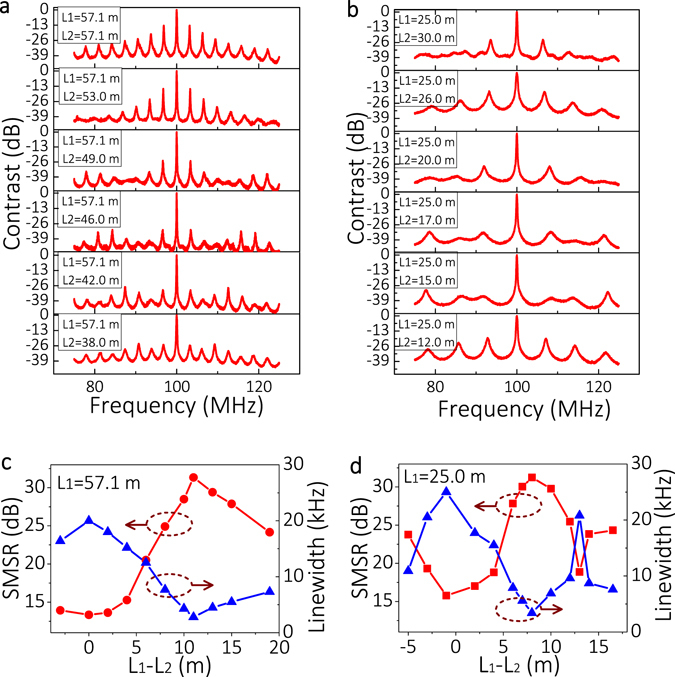
(**a**,**b**) Detected normalized power spectra for selected different combinations of feedback lengths with DCFS. (**c**,**d**) The overall SMSR (red points) from the detected normalized power spectra and their corresponding linewidth (blue points) from the SCELD measurements.

To find two suitable feedback lengths in the DCFS, we fixed a constant feedback length (L_1_) and changed the other feedback length (L_2_), whose length is near L_1_ because small value of Δ*L* would lead to large FSR and ease to achieve SLM according to Vernier principle. Figure 5(a) and (b) shows the evolution of the normalized power spectra when varying L_2_ and fixing L_1_ = 57.1 and 25.0 m, respectively. Figure 5(c) and (d) shows the corresponding SMSR (red points) and linewidth (blue points).

When L_1_ and L_2_ are nearly identical, the DCFS should has a large FSR to obtain a high SMSR according to the Vernier principle. However, the experimental results show relative small SMSRs around ΔL = 0. This is because the cavity mode usually has a certain bandwidth to produce mode overlapping in two cavities. The overlapped modes will finally become side modes and prevent the improvement of SMSR. When ΔL is increased, the mode overlapping is suppressed in the two cavities, and hence the SMSR increases and the linewidth is gradually compressed. When ΔL arrives at a certain value, e.g. ΔL = 11.1 m with L_1_ = 57.1 m in Fig. 5(c) and ΔL = 8 m with L_1_ = 25 m in Fig. 5(d), we achieved a SLM DFB laser with a large SMSR of around 31 dB. If we further increased ΔL, the mode overlapping is increased to present more side modes in the normalized power spectra, hence the SMSR deteriorates again.

When the side modes are efficiently suppressed and we achieve a high SMSR, the laser linewidth was naturally compressed as it obtained more gain at the main lasing mode. Therefore, we simultaneously realized the SMSR improvement and linewidth compression. For instance, the linewidth was compressed to 2.8 kHz with a SMSR of 31.34 dB when L_1_ = 57.1 m and L_2_ = 46.0 m, and the linewidth was compressed to 3.0 kHz with a SMSR of 31.15 dB when L_1_ = 25.0 m and L_2_ = 17 m.

**Figure 6:**
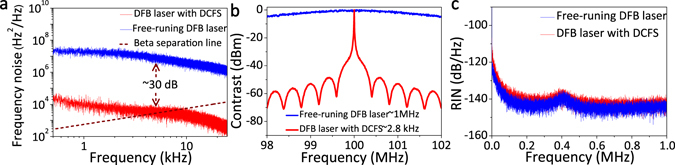
(**a**) Frequency-noise power spectral density of free-running DFB laser (blue line) and DFB laser with DCFS (red line), and the β-separation line given by 8ln(2)f/π2. (**b**) Normalized power spectrum of free-running DFB laser (blue line) and DFB laser with DCFS (red line). (**c**) Relative intensity noise (RIN) of free-running DFB laser (blue line) and DFB laser with DCFS (red line).

Figure 6 shows the frequency noise characteristics of the output laser with L_1_ = 57.1 m and L_2_ = 46.0 m. The measurement system of frequency-noise power spectral density (PSD) has been described by Li *et al*.^26^. The frequency-noise PSD of the DFB laser with DCFS can be suppressed by about 30 dB in comparison to the free-running DFB laser (see Fig. 6(a)). From the β-separation line shown in Fig. 6(a), the output laser linewidth with DCFS can be estimated to be about a few kilohertz^27^. We used the SCELD method with 500 m delaying fiber to measure the linewidth more accurately, and it is found that the laser linewidth is compressed from ~1 MHz to 2.8 kHz when L_1_ = 57.1 m and L_2_ = 46.0 m.

The output power stability is an important parameter for the laser. Figure 6(c) illustrates the relative intensity noise (RIN) performance of the laser from 0~1 MHz with 0.25 mW detected power by adding a variable optical attenuator. In the case of the free-running DFB laser, the DFB RIN is lower than ~145 dB/Hz when the frequency is larger than 500 kHz and the RIN level of the relaxation oscillation peak of DFB is ~−137 dB/Hz at ~400 kHz. The RIN level of the DFB laser with DCFS is ~3 dB/Hz larger than the free-running DFB laser. A power meter was also used to monitor the output port of the 1 × 3 coupler, measuring the output power to be ~5.955 dBm and the stability of the laser output was found to vary less than 0.001 dBm without cavity feedback and, 0.009 dBm with DCFS, respectively. So the stability of the output power is ~0.21% in a DCSF structure. The stability of the output power deteriorated when the number of feedback cavities was increased, as more external noise was injected into the DFB. Furthermore, if we increase to three-cavity or four-cavity, a 1 × 4 coupler or a 1 × 5 coupler has to be used to connect more feedback cavities, which will decrease both the output power and the feedback rate. Hence, more feedback cavities are also not conducive to compressing laser linewidth. Therefore, to obtain a stable narrow linewidth laser, it is not necessary to further increase the number of the feedback cavities once the SLM laser has been achieved.

**Figure 7:**
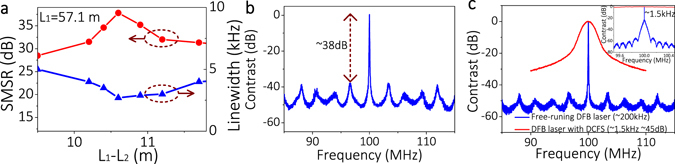
(**a**) SMSRs and linewidths with finely tuned feedback length. (**b**) Output power spectrum when L_1_ = 57.1 m L_2_ = 46.5 m. (**c**) Output power spectra of 200 kHz laser linewidth (blue curve) with or without DCFS (red curve). Inset: magnified power spectra in a small frequency span of 1 MHz.

From Fig. 5(a) and (c), a SMSR of 31.34 dB has been achieved when two feedback lengths were coarsely chosen to be L_1_ = 57.1 m and L_2_ = 46.0 m. In order to achieve higher SMSR, we finely tuned the feedback length of L_2_ near 46.0 m and fixed the feedback length of L_1_, as shown in Fig. 7(a). Finally, a SMSR of 38 dB was achieved at L_1_ = 57.1 m and L_2_ = 46.5 m as shown in Fig. 7(b), and the laser linewidth was compressed to ~2.7 kHz.

In order to demonstrate the wide applicability of our proposed scheme, we tested the performance of another DFB laser by using the same DCFS above. This DFB laser works at the wavelength of 1550.12 nm with a linewidth of 200 kHz. By using the DCFS with L_1_ = 57.1 m and L_2_ = 46.5 m, the SMSR was greatly enhanced to 45 dB and the linewidth was compressed to ~1.5 kHz, as shown in Fig. 7(c). Here, the linewidth was measured by using the SCELD method with a 2 km delaying fiber.

The scheme is wavelength independent as long as the feedback structure operation. Narrower linewidth and more stability of the output laser would be obtained by using an isolation package of the feedback structure, polarization maintaining feedback or optimizing the output isolation (γ) of DFB laser. The feedback structure would be also replaced by FBG or other filters with tuneable reflectivity to simplify the feedback structure and increase the output power, but the coverage of the wavelength by FBG or other filters is much smaller than this feedback structure with an optical coupler and an optical circulator, so it is inconvenient for tuneable wavelength laser linewidth compression to utilize FBG or other filters as the feedback structure because of its wavelength selectivity.

## Conclusions

In conclusion, a simple DCFS was introduced for linewidth compression of a commercial DFB laser. The SCELD method was used to monitor the evolution of output laser linewidth under different conditions. Experimental results demonstrated that laser linewidthes of ~1 MHz and ~200 kHz were successfully compressed to ~2.7 and ~1.5 kHz with a side mode suppression ratio of 38 and 45 dB, respectively, by using DCFS. The proposed DCFS can also be applied in distributed Bragg reflector fiber laser, Fabry-Pérot laser diode (FP-LD) or other high finesse optical feedback systems to further compress laser linewidth.

## Methods

### Linewidth measurement method

The power spectra of the output laser under different conditions were monitored by the detection system (see Figs 1 and 2), then an accurate laser linewidth was determined from a fit to the detected power spectrum by SCELD method^22, 23^. The acoustic optical modulator (AOM: 100 MHz, Gooch & Housego) was used to generate a frequency shift. The combining laser was monitored by a photoelectric detector (PDB430C, 350 MHz, Thorlabs). The power spectrum signal from PD was obtained with an electric spectrum analyzer (FSV, 10 Hz–30 GHz, ROHDE&SCHWARZ). The principle of this experimental setup and the detected process could also be found in the refs 22 and 23, respectively.

### Frequency noise and RIN measurement method

The frequency noise of the output laser was detected with an optical phase demodulator system (OPD-4000) manufactured by Optiphase Inc., and the RIN of the output laser was measured by the InGaAs photodetector (PDA10CF, 150 MHz, Thorlabs) and the ESA (FSV, 10 Hz–30 GHz, ROHDE&SCHWARZ).
